# SARS-CoV-2 Infection of Microglia Elicits Proinflammatory Activation and Apoptotic Cell Death

**DOI:** 10.1128/spectrum.01091-22

**Published:** 2022-05-05

**Authors:** Gi Uk Jeong, Jaemyun Lyu, Kyun-Do Kim, Young Cheul Chung, Gun Young Yoon, Sumin Lee, Insu Hwang, Won-Ho Shin, Junsu Ko, June-Yong Lee, Young-Chan Kwon

**Affiliations:** a Center for Convergent Research for Emerging Virus Infection, Korea Research Institute of Chemical Technology, Daejeon, Republic of Korea; b Arontier Co., Ltd., Seoul, Republic of Korea; c Department of Predictive Toxicology, Korea Institute of Toxicology, Daejeon, Republic of Korea; d Department of Microbiology and Immunology, College of Medicine, Yonsei University, Seoul, Republic of Korea; e Institute for Immunology and Immunological Disease, Brain Korea 21 Project for Medical Science, Yonsei University College of Medicine, Seoul, Korea; Karolinska Institutet

**Keywords:** SARS-CoV-2, microglia, neuroinflammation, M1 polarization, apoptosis

## Abstract

Accumulating evidence suggests that severe acute respiratory syndrome coronavirus 2 (SARS-CoV-2) infection causes various neurological symptoms in patients with coronavirus disease 2019 (COVID-19). The most dominant immune cells in the brain are microglia. Yet, the relationship between neurological manifestations, neuroinflammation, and host immune response of microglia to SARS-CoV-2 has not been well characterized. Here, we reported that SARS-CoV-2 can directly infect human microglia, eliciting M1-like proinflammatory responses, followed by cytopathic effects. Specifically, SARS-CoV-2 infected human microglial clone 3 (HMC3), leading to inflammatory activation and cell death. RNA sequencing (RNA-seq) analysis also revealed that endoplasmic reticulum (ER) stress and immune responses were induced in the early, and apoptotic processes in the late phases of viral infection. SARS-CoV-2-infected HMC3 showed the M1 phenotype and produced proinflammatory cytokines, such as interleukin (IL)-1β, IL-6, and tumor necrosis factor α (TNF-α), but not the anti-inflammatory cytokine IL-10. After this proinflammatory activation, SARS-CoV-2 infection promoted both intrinsic and extrinsic death receptor-mediated apoptosis in HMC3. Using K18-hACE2 transgenic mice, murine microglia were also infected by intranasal inoculation of SARS-CoV-2. This infection induced the acute production of proinflammatory microglial IL-6 and TNF-α and provoked a chronic loss of microglia. Our findings suggest that microglia are potential mediators of SARS-CoV-2-induced neurological problems and, consequently, can be targets of therapeutic strategies against neurological diseases in patients with COVID-19.

**IMPORTANCE** Recent studies reported neurological and cognitive sequelae in patients with COVID-19 months after the viral infection with several symptoms, including ageusia, anosmia, asthenia, headache, and brain fog. Our conclusions raise awareness of COVID-19-related microglia-mediated neurological disorders to develop treatment strategies for the affected patients. We also indicated that HMC3 was a novel human cell line susceptible to SARS-CoV-2 infection that exhibited cytopathic effects, which could be further used to investigate cellular and molecular mechanisms of neurological manifestations of patients with COVID-19.

## INTRODUCTION

Coronaviruses, which are enveloped positive-single-stranded RNA viruses that belong to the *Coronaviridae* family, cause mild to severe respiratory, enteric, and neurological diseases in humans and animals (1). At the end of 2019, a pneumonia outbreak caused by severe acute respiratory syndrome coronavirus 2 (SARS-CoV-2) was reported in Wuhan, China, and this novel coronavirus gave rise to the current coronavirus disease 2019 (COVID-19) pandemic (2). By cross-species transmission to humans, as of December 2021, over 280 million COVID-19 confirmed cases and 5 million deaths globally have been reported, according to COVID-19 situation reports from WHO (https://www.who.int/emergencies/diseases/novel-coronavirus-2019/situation-reports).

Although coronavirus disease 2019 (COVID-19) is primarily characterized as a respiratory disease, multiple organ dysfunction syndromes may occur in several organs, including the brain, which contributes to neurological manifestations (3). Acute neurological and psychiatric complications of COVID-19 often occurred even in persons younger than 60 years of age (4). Moreover, cortical signal alteration (5), loss of white matter, and axonal injury (6) have been reported, as well as increasing observations of neurological issues, including headache, ischemic stroke, seizures, delirium, anosmia, ageusia, encephalopathy, and total paralysis (7–14). Patients with more severe infections are more likely to have neurological manifestations and impairment and are at a higher risk of mortality (15).

Microglia are macrophage-like brain immune cells in the central nervous system (CNS). They have key functions in maintaining brain homeostasis and in the rapid response to injury and inflammation (16). When microglia respond to immunological stimuli, they become activated and transform from a ramified into an amoeboid morphology, releasing interleukin (IL)-1β, IL-6, and tumor necrosis factor-α (TNF-α) (17). Activated microglia consist of a dual phenotype, wherein M1, or the classically activated state, is neurotoxic and involved in neuroinflammation, and M2, or the alternatively activated state, is neuroprotective (18–20). Increasing evidence suggests that the overactivation and dysregulation of microglia might result in disastrous and progressive neurotoxic consequences (21–24). In the brains of deceased COVID-19 patients, microgliosis and immune cell accumulation were observed (25), as well as microglial nodules caused by massive microglial activation in the medulla oblongata (26) and cerebellar dentate nuclei (27). The neuroinvasive capacity (28) and olfactory transmucosal invasion of SARS-CoV-2 in patients with COVID-19 (29) have also been reported. Additionally, human microglia express SARS-CoV-2 entry factors, such as angiotensin-converting enzyme 2 (ACE2) and transmembrane protease serine subtype 2 (TMPRSS2) (30). Thus, we hypothesized that microglial activation by direct SARS-CoV-2 infection could be one of the major mechanisms driving the neuroinflammation and neurological complications.

Despite accumulating evidence, little is known regarding the mechanisms involved in the neuroinflammation of SARS-CoV-2 infection. In this study, we demonstrated that SARS-CoV-2 can directly infect human microglia and induce proinflammatory responses, reflecting polarization toward the M1 phenotype. We further showed that the SARS-CoV-2 infection led to apoptosis as a cytopathic effect (CPE) through both intrinsic and extrinsic apoptotic pathways. Moreover, we found that murine microglia expressing human ACE2 (hACE2) were infected by intranasally administered SARS-CoV-2, followed by microglial proinflammatory activation and loss of their population.

## RESULTS

### SARS-CoV-2 directly infects a human microglial cell line with cytopathic effects.

**FIG 1:**
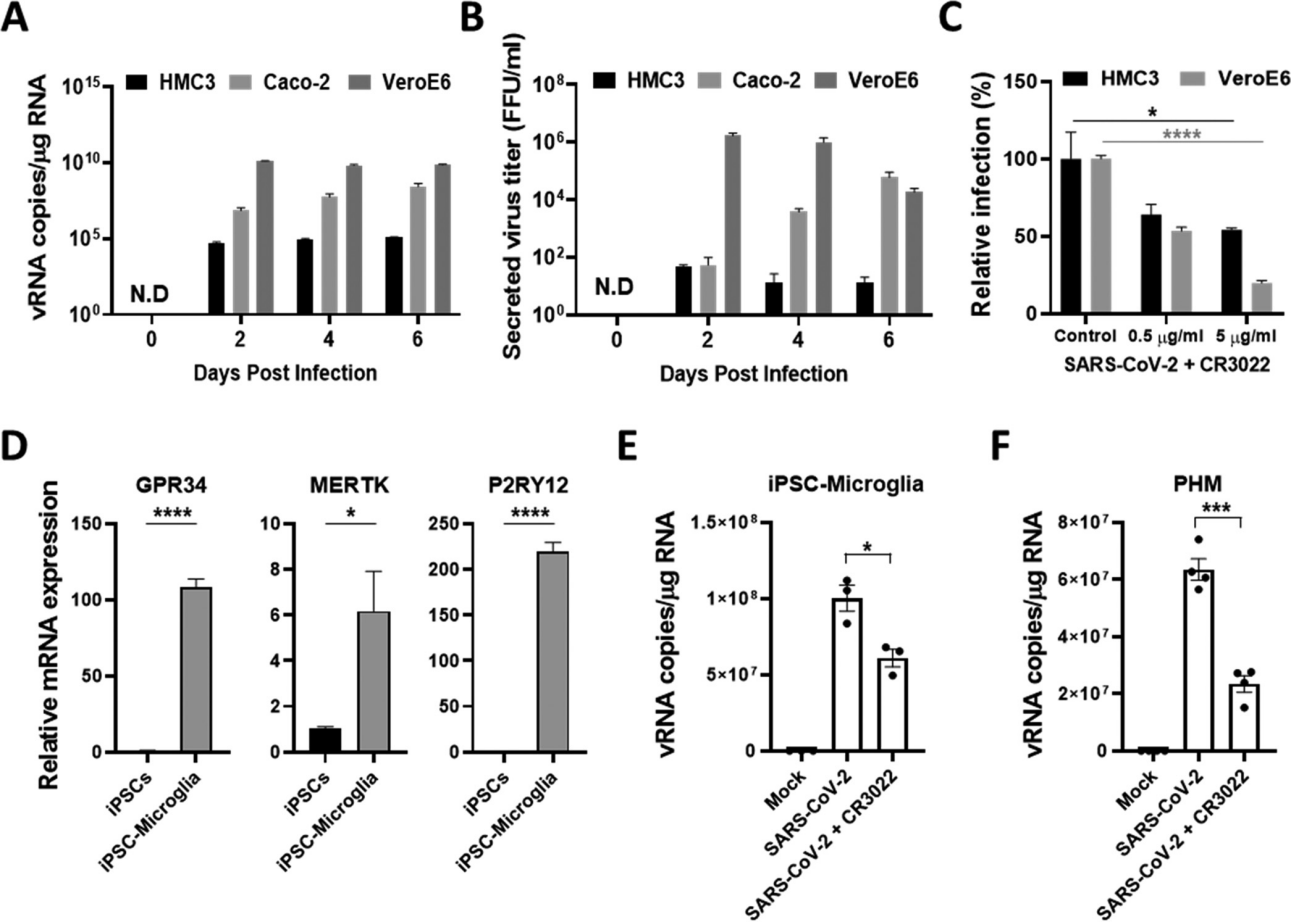
SARS-CoV-2 directly infects human microglia cells. (A) HMC3, Caco-2, and Vero E6 cells were infected with one MOI of SARS-CoV-2. The total cellular RNA was extracted at 2, 4, and 6 dpi to detect the viral RNA of the SARS-CoV-2 NP gene by quantitative real-time PCR (RT-qPCR). The graph shows viral RNA copies per microgram of total cellular RNA each day. (B) The culture media derived from SARS-CoV-2-infected cells were serially diluted and used for focus forming assay. The graph shows the secreted virus titer as focus forming units (FFU). (C) The graph shows viral RNA copies per microgram of total cellular RNA at 2 dpi after treatment with the increasing amount of CR3022 neutralizing antibody. (D) RT-qPCR analysis of the expression of microglial signature genes (GPR34, MERTK, and P2RY12) in iPSC and iPSC-Microglia for the characterization of iPSC-Microglia. (E and F) Viral RNA levels of SARS-CoV-2-infected iPSC-Microglia (E) and PHM (F) in the absence/presence of CR3022 neutralizing antibody at 1 dpi. Statistically significant differences between the groups were determined by one-way analysis of variance (ANOVA; C) and Student's *t* test (D, E, F); *, *P* < 0.05; ***, *P* < 0.001; ****, *P* < 0.0001. Symbols represent mean ± SEM.

**FIG 2:**
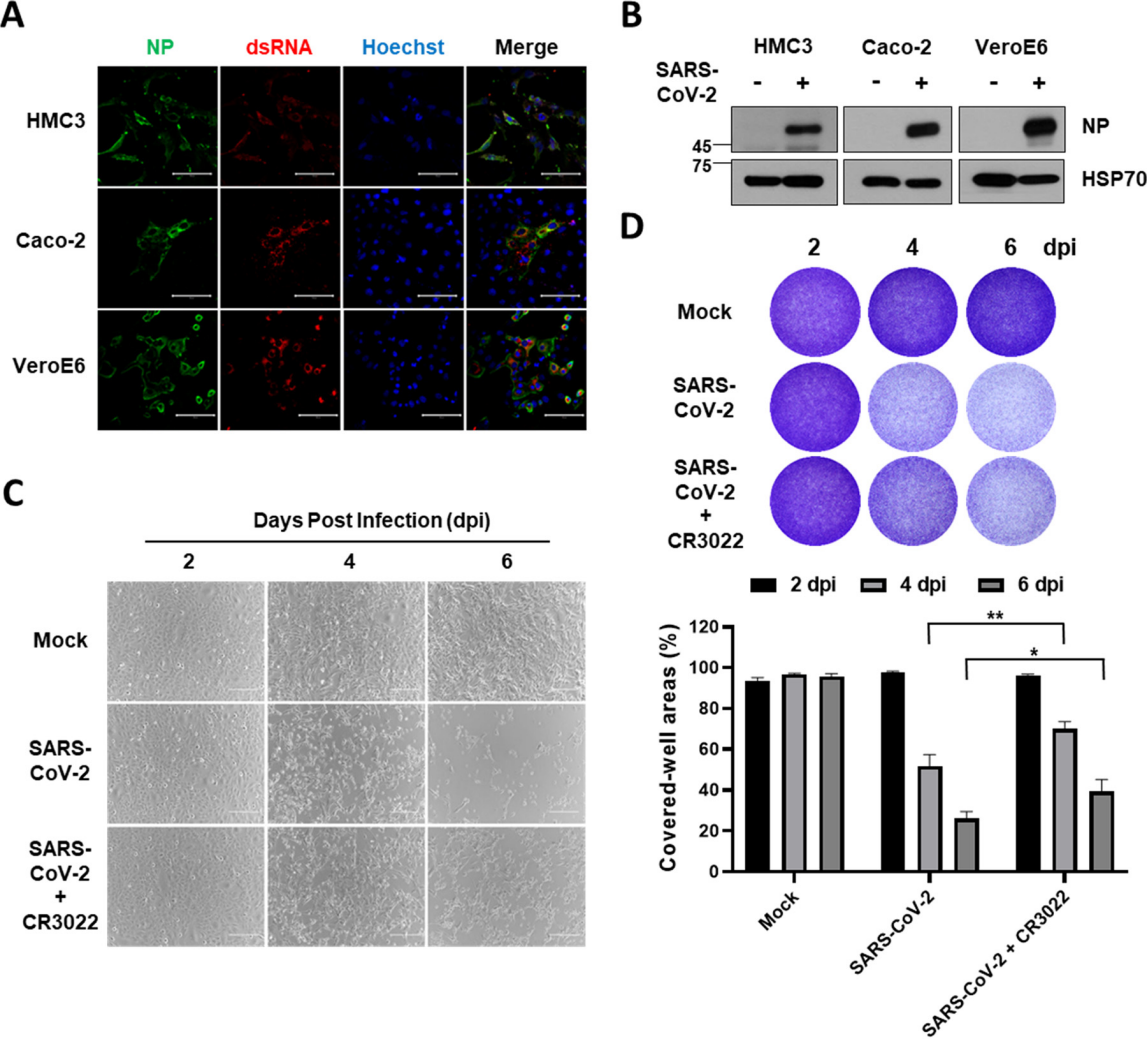
SARS-CoV-2 infection of human microglia cells elicits the cytopathic effects (CPE). (A) Confocal images of SARS-CoV-2-infected HMC3 (top row), Caco-2 (middle row), and Vero E6 (bottom row) demonstrate infection of these cells by immunofluorescence assay with anti-SARS-CoV-2 NP and anti-dsRNA antibodies. Scale bar = 100 μm. (B) Western blotting of SARS-CoV-2 NP in each infected cell. The 70-kDa heat shock protein (Hsp70) served as the loading control. (C) Phase-contrast images of the mock or SARS-CoV-2-infected HMC3 in the absence/presence of CR3022 neutralizing antibody at 2, 4, and 6 dpi, indicating cell death as the CPE by microscopy. Scale bar = 200 μm. (D) Images of crystal violet staining of the mock or SARS-CoV-2-infected HMC3 in the absence/presence of CR3022 neutralizing antibody, plated in the 12-well (upper). The graph shows the percent measurements of crystal violet-stained cell covered areas by the ImmunoSpot reader (lower). Statistically significant differences between the groups were determined by Student's *t* test; *, *P* < 0.05; **, *P* < 0.01. Symbols represent mean ± SEM.

In a postmortem study, more than half of the patients with COVID-19 demonstrated high microglial immune activation with microglial nodules, and immune filtration was linked to axonal damage (31). Another study reported that viral nucleocapsid protein (NP) was localized into microglia of the brain cortex in deceased COVID-19 patients (32). To investigate whether SARS-CoV-2 can infect human microglia, we used immortalized human embryonic brain-derived primary microglia (HMC3). This cell line was infected with one multiplicity of infection (MOI) of SARS-CoV-2, along with other well-known SARS-CoV-2 susceptible cell lines, including Caco-2, which is derived from human colon carcinoma, and Vero E6, which is a kidney epithelial cell line from an African green monkey (33). Viral RNA was detected by quantitative real-time PCR (RT-qPCR) and was slightly increased in HMC3 in a time-dependent manner, despite the lower viral copies per microgram of the total RNA than that of Caco-2 and Vero E6 (Fig. 1A). When secreted virus particles were measured by focus forming assay, the progeny viruses were detected in line with intracellular viral RNA at a much lower level than that of Caco-2 and Vero E6 (Fig. 1B). To validate the viral infection, we used a CR3022 neutralizing antibody to block the infection (34). Notably, the relative infection percentages of both HMC3 and Vero E6 were significantly reduced at 2 days postinfection (dpi) by CR3022 neutralizing antibody in a dose-dependent manner (Fig. 1C). To assess the susceptibility more accurately to SARS-CoV-2 infection, we introduced both human microglia derived from human induced pluripotent stem cells (iPSC-Microglia) and commercially available primary human brain microglial cells (PHM). The iPSC-Microglia were identified by assessing the expression of microglial signature genes, such as G protein-coupled receptor 34 (GPR34), MER proto-oncogene, and tyrosine kinase (MERTK), and Purinergic receptor P2Y12 (P2RY12) (35) (Fig. 1D). Both cells were inoculated with one MOI as described above in the absence/presence of 5 μg/mL CR3022. The viral RNA levels of both cells were higher than those of HMC3 and significantly reduced by the neutralization (Fig. 1E and F). To confirm that SARS-CoV-2 infects HMC3, the three cell lines were infected as before, followed by an immunofluorescence assay to detect dsRNA and NP at 3 dpi, verified by an additional detection of S and NP (Fig. 2A and Fig. S1). In addition, the cell lysates at 3 dpi were analyzed by Western blotting to detect NP (Fig. 2B). Importantly, SARS-CoV-2 infection of HMC3 triggered cell death as one of the CPEs (Fig. 2C), similar to that in Vero E6, which has been known to exhibit CPEs, but not Caco-2 (33) (Fig. S2). Beginning at 4 dpi, infected HMC3 led to cell death, and by 6 dpi, most cells had died. Crystal violet staining at 4 and 6 dpi showed that the cell-covered areas were diminished but those of 4 and 6 dpi were significantly recovered by CR3022 neutralizing antibody (Fig. 2D). Thus, these findings proposed that HMC3 is another human cell line that is susceptible to SARS-CoV-2 infection and exhibits CPEs.

### Distinct transcriptional signatures and gene expression changes in SARS-CoV-2-infected HMC3.

**FIG 3:**
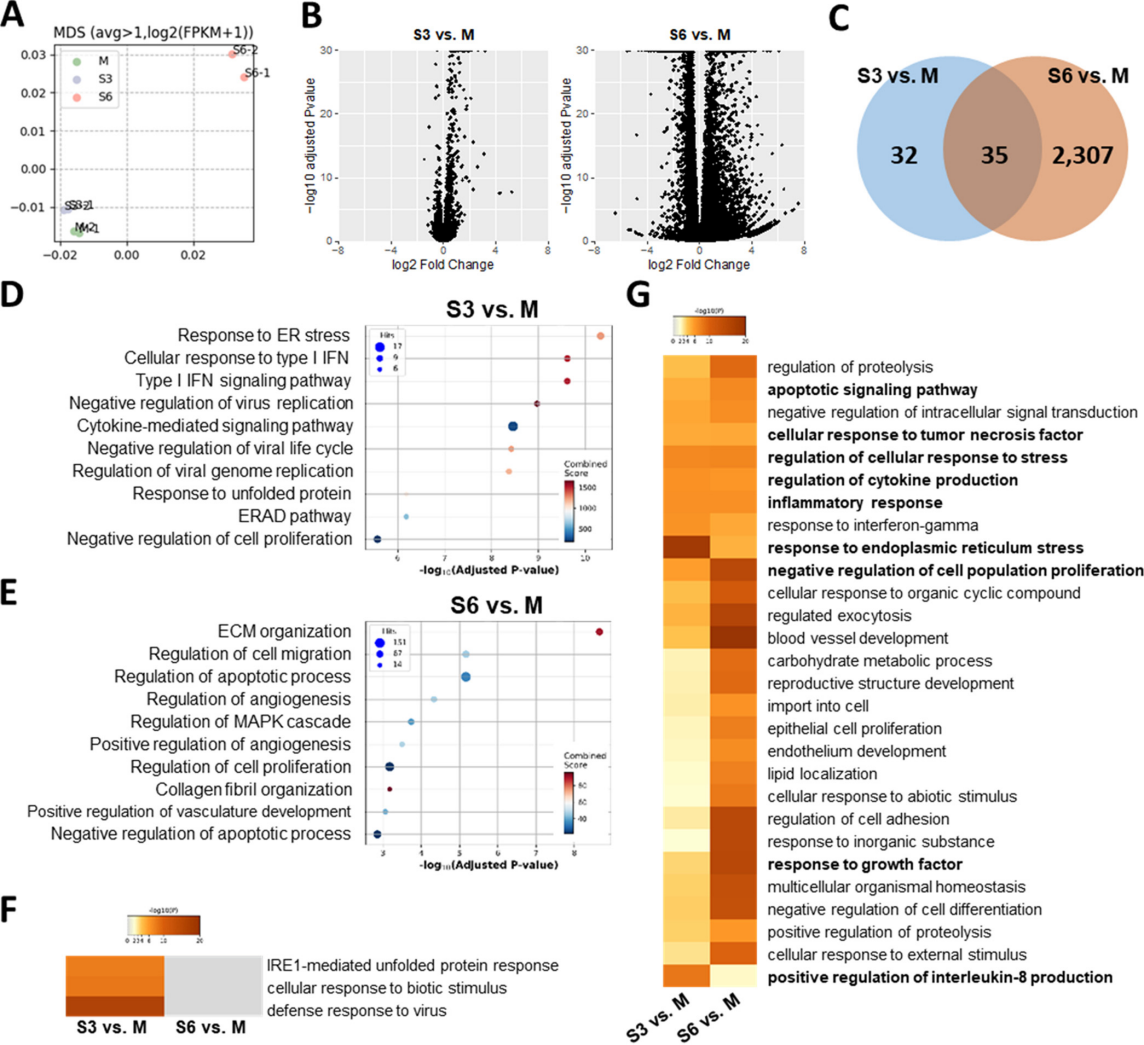
RNA-sequencing analysis of SARS-CoV-2-infected HMC3 cells. (A) Multidimensional analysis of genes expressed over one mean fragment per kilobase per million mapped fragments (FPKM). (B) Volcano plots of SARS-CoV-2-infected HMC3 cells at 3 dpi (S3; left) and 6 dpi (S6; right) compared to mock (M). (C) Venn diagram of differently expressed genes (DEGs) at S3 and S6. (D and E) Top 10 gene ontology (GO) enrichment terms for (D) S3 and (E) S6 DEGs. (F and G) Heatmaps showing GO enrichment terms for S3 and S6 significantly changed in both (F) S3 and (G) S6.

To assess the effect of SARS-CoV-2 infection on gene expression in HMC3, we performed RNA-seq analysis on SARS-CoV-2-infected HMC3 at 3 and 6 dpi (S3 and S6, respectively), and an uninfected control (Mock, M), for comparison. Overall, the gene expression data indicated that M and S3 had relatively similar transcriptional signatures, while S6 had different signatures both in fold change, and adjusted *P* value (Fig. 3A and B). Differentially expressed gene (DEG) counts largely increased from 67 (S3) to 2,342 (S6) (Fig. 3C). More than half of the S3 DEGs overlapped (52.2%, 35/67 DEGs) with S6, and most of these genes were upregulated both at S3 and S6 (88.57%, 31/35 overlapped DEGs) (Fig. S3A). These results suggest that gene expression changes induced by SARS-CoV-2-infection increased with time, and accelerated more from S3 to S6 than from M to S3.

According to the overrepresentation analysis (ORA) of the gene ontology (GO) biological process, S3 DEGs in the early phase of viral infection were highly enriched for endoplasmic reticulum (ER) stress conditions, and antiviral immune responses, including type I interferons (IFNs) and cytokine-mediated signaling pathways (Fig. 3D). Given that RNA viruses induce ER stress through viral polypeptides and immune responses by double-stranded-RNA intermediates (36, 37), this result would imply SARS-CoV-2 infection and replication in HMC3. On the other hand, S6 DEGs were highly enriched for apoptotic processes (Fig. 3E). Although ER stress-related terms were not listed in Fig. 2E, most of the upregulated DEGs in both S3 and S6 (DNAJB7, DDIT3, HSPA5, HSP90B1, HYOU1, PDIA4, SEL1L) were highly enriched for ER stress, indicating that it is still induced at S6 (Fig. S3B). Given that prolonged ER stress causes apoptosis (38), these results imply that defense responses against SARS-CoV-2 infection are elicited in the early phase of the viral infection, with later gene expression changes contributing to apoptosis. In line with this, heatmap and network analyses of GO biological processes using Metascape (Fig. 3F and G and Fig. S4), immune responses, cytokine production, ER stress responses, and defense responses to the virus were distinctly enriched in S3, while apoptotic signaling pathways, negative regulation of cell population, and growth factor response were significantly altered in S6. To conclude, SARS-CoV-2 infection in HMC3 induced gene expression of antiviral immune and ER stress responses in the early phase (S3) and apoptosis in the late phase of infection (S6).

### Microglial proinflammatory activation and M1 phenotype polarization by SARS-CoV-2-infection.

**FIG 4:**
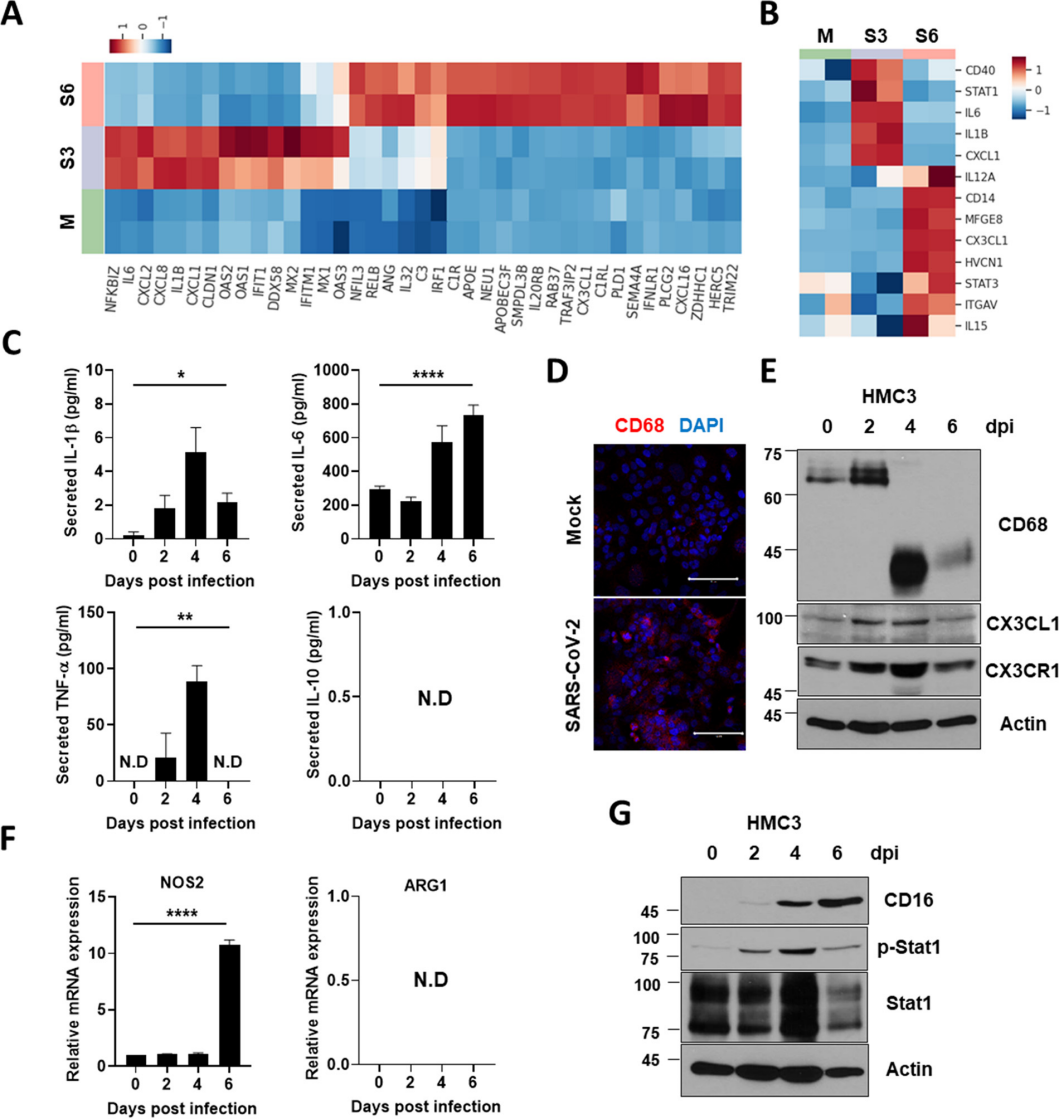
Proinflammatory activation and M1 polarization of HMC3 by SARS-CoV-2 infection. (A and B) Heat maps of significantly upregulated genes during SARS-CoV-2 infection enriched in (A) immune response and (B) microglial M1 polarization. (C) The graphs show the measurements of secreted proinflammatory cytokines, including IL-1β, IL-6, and TNF-α, and anti-inflammatory cytokine IL-10 by enzyme-linked immunosorbent assay (ELISA). (D) Confocal immunofluorescence images of mock or SARS-CoV-2-infected HMC3 with anti-CD68 antibody. (E) Quantitative analysis of microglial activation markers, including CD68, CX3CL1, and CX3CR1 using Western blotting. Actin served as the loading control. (F) RT-qPCR analysis of the representative M1 (NOS2; left) and M2 markers (Arginase-1; right). (G) Assessment of proteins of M1 markers, including CD16, phopho-Stat1, and Stat1 by Western blotting. Actin served as the loading control. Statistically significant differences between the groups were determined using ANOVA; *, *P* < 0.05; **, *P* < 0.01; ****, *P* < 0.0001. Symbols represent mean ± SEM.

In response to viral infection, microglia are activated and polarized into the proinflammatory M1 phenotype or the anti-inflammatory M2 phenotype (39). To assess the effects of SARS-CoV-2 infection on microglial activation and polarization in HMC3, we analyzed the DEGs associated with immune response and microglial polarization. Several genes related to type I IFNs and innate immunity, such as MXs, the 2’,5′-oligoadenylate synthetases (OASs) and complement component 3 (C3), were upregulated at 3 or 6 dpi in the RNA-seq data (Fig. 4A). The M1 phenotype polarization-related genes, such as IL-1β, IL-6, and CXCL1 also showed increased RNA expression levels (Fig. 4B), indicating that SARS-CoV-2 evoked proinflammatory activation and polarization toward the M1 phenotype in HMC3. To confirm this, we assessed the expression of ILs and IFNs by RT-qPCR (Fig. S5) and detected secreted cytokines in culture media by enzyme-linked immunosorbent assay (ELISA) (Fig. 4C). Pro-inflammatory cytokines, such as IL-1β, IL-6, and TNF-α, but not the immune regulatory cytokine IL-10, were highly produced and secreted due to SARS-CoV-2 infection. CD68 is a lysosomal protein expressed in high levels by macrophages and activated microglia and in low levels by resting microglia (40). The protein expression level of CD68 was increased in infected HMC3 (Fig. 4D), along with other markers of microglial proinflammatory activation, such as CX3CL1 and CX3CR1 (Fig. 4E). These results indicate that SARS-CoV-2-infected HMC3 are inflammation-activated. To verify whether activated microglia polarized into the M1 phenotype, we estimated the RNA and protein expression of the M1 phenotype markers. When we assessed the RNA expression level of each representative marker of the M1 (NOS2) and M2 phenotypes (Arginase-1), only NOS2 was highly expressed at 6 dpi (Fig. 4F). Protein expression levels of M1 markers, CD16, phospho-Stat1, and Stat1, were also induced at 4 and 6 dpi (Fig. 4G). Therefore, by SARS-CoV-2 infection, HMC3 was activated and polarized into the inflammatory M1 phenotype, producing proinflammatory cytokines.

### Intrinsic and extrinsic death-receptor mediated apoptotic cell death by SARS-CoV-2 infection.

**FIG 5:**
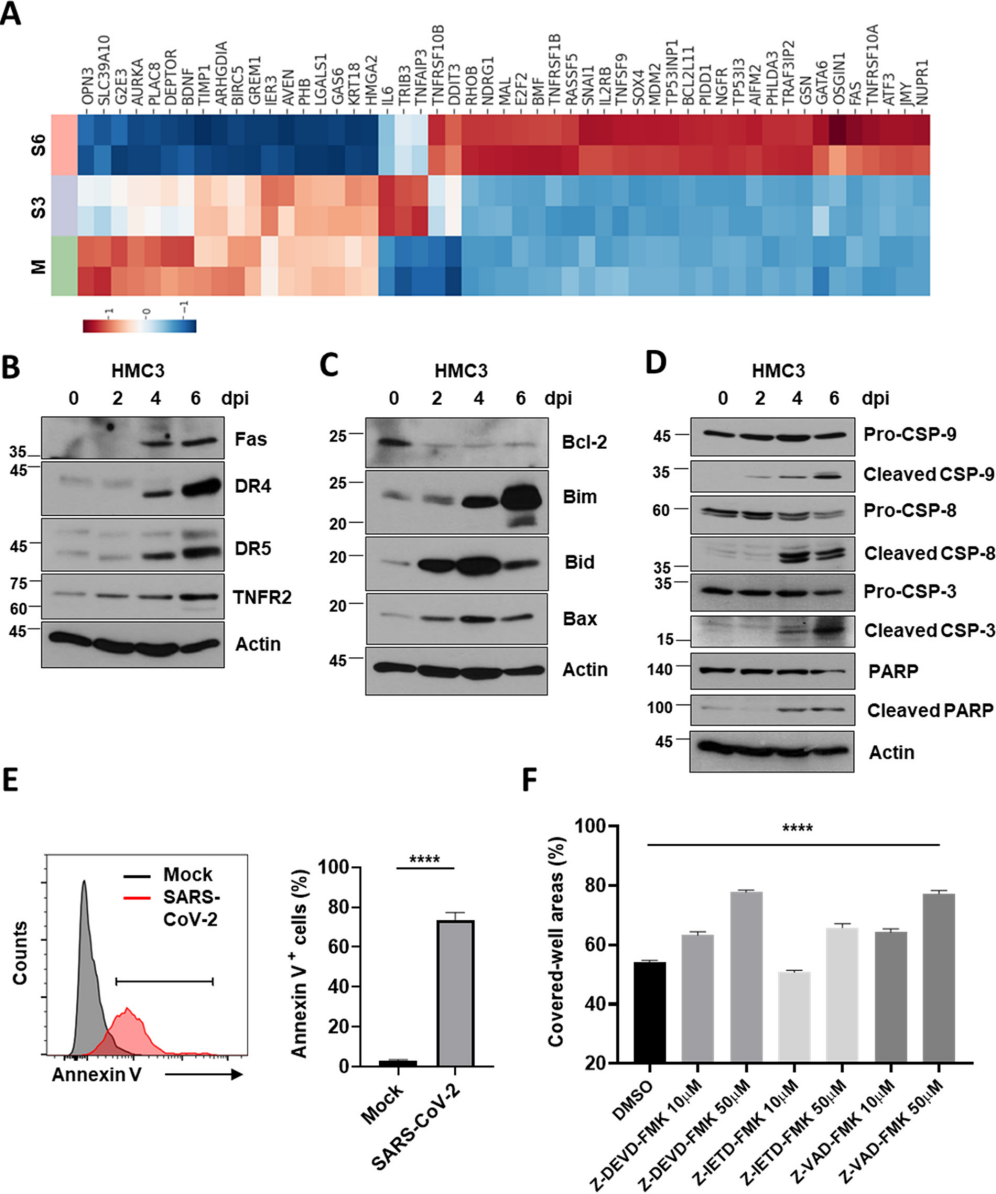
Intrinsic and death receptor (DR)-mediated extrinsic apoptosis in SARS-CoV-2-infected HMC3. (A) A heat map of significantly upregulated and downregulated genes during SARS-CoV-2 infection enriched in the apoptotic process. (B to D) Extrinsic apoptosis-related proteins, including Fas, Death receptors (DRs), and tumor necrosis factor receptor 2 (TNFR2) (B); intrinsic apoptosis-associated proteins, such as Bcl-2, Bim, Bid, and Bax (C); and caspases and poly (ADP-ribose) polymerase (PARP) (D), which are downstream of both intrinsic and extrinsic apoptosis, were quantitatively analyzed using Western blotting. Actin served as the loading control. (E) At 3 d postinfection (dpi), the cell surface of the mock or SARS-CoV-2-infected HMC3 was bound with recombinant human Annexin V to detect cells that are in progress of apoptosis by flow cytometry analysis. The histogram peaks indicate mock (gray) and infected (red) cells (left). The percentage of Annexin V positive cells is shown in the bar graph (right). (F) The infected HMC3 cells were treated with Z-DEVD-FMK (caspase-3 inhibitor), Z-IETD-FMK (Caspase-8 inhibitor), and Z-VAD-FMK (pan-caspase inhibitor) for 6 days at the indicated concentrations. The surviving cells were stained with crystal violet and then the percent measurements of the stained cell covered areas were obtained using an ImmunoSpot reader. Statistically significant differences between the groups were determined by Student's *t* test (E) or one-way ANOVA (F); ****, *P* < 0.0001. Symbols represent means ± SEM.

As illustrated in Fig. 2, SARS-CoV-2 infection leads to cell death in HMC3. Several viruses, such as the human immunodeficiency virus-1 (HIV-1), hepatitis C virus (HCV), and human papillomavirus (HPV), activate death receptor (DR)-mediated apoptosis in different ways (41). To address the mechanism of cell death caused by SARS-CoV-2, we thought that infected cells would die through apoptosis, which includes three major ways of programmed cell death (PCD), such as necrosis and pyroptosis. The expression of several genes associated with apoptotic processes in DEGs was altered toward that of pro-apoptosis. Representatively, key regulator genes of cell cycle progression and cell proliferation, such as Aurora kinase A (AURKA) and Baculoviral Inhibitor of apoptosis repeat-containing 5 (BIRC5), were downregulated. On the other hand, apoptosis-associated and death genes, like DNA damage-inducible transcript 3 (DDIT3), RHOB, FAS, and p53-induced death domain protein 1 (PIDD1) were substantially upregulated (Fig. 5A). According to the Western blot of proteins related to the apoptotic process, not only extrinsic death-receptor mediated proteins (Fig. 5B) but also those of the intrinsic pathways (Fig. 5C) of apoptosis were elicited in the infected HMC3. Expression of death receptors, such as Fas, DR4, DR5, and TNF receptor 2 (TNFR2), which initiate extrinsic apoptosis, were augmented at 4 and 6 dpi. On the other hand, the expression of an apoptosis regulator, Bcl-2, which suppresses apoptosis, was reduced, and those of Bim, Bid, and Bax, which are essential for the activation of BAX-dependent PCD, were increased. Cleavage of caspase-9 in the intrinsic pathway, caspase-8 in the extrinsic pathway, caspase-3, and poly (ADP-ribose) polymerase (PARP) were observed, demonstrating both pathways of apoptosis (Fig. 5D). Death receptor-mediated apoptosis represents an efficient mechanism by which the virus can induce cell death (41), and ER stress induced by viral infection is mainly involved in the intrinsic pathway (36, 37). Elicited apoptosis in the infected HMC3 was confirmed by Annexin V staining (Fig. 5E). To confirm apoptosis, we measured the surviving cells at 6 dpi via crystal violet staining following the treatment of potent and selective inhibitors against caspases. Z-DEVD-FMK (caspase-3 inhibitor), Z-IETD-FMK (caspase-8 inhibitor), and Z-VAD-FMK (pan-caspase inhibitor) treatments significantly blocked apoptotic cell death (Fig. 5F).

**FIG 6:**
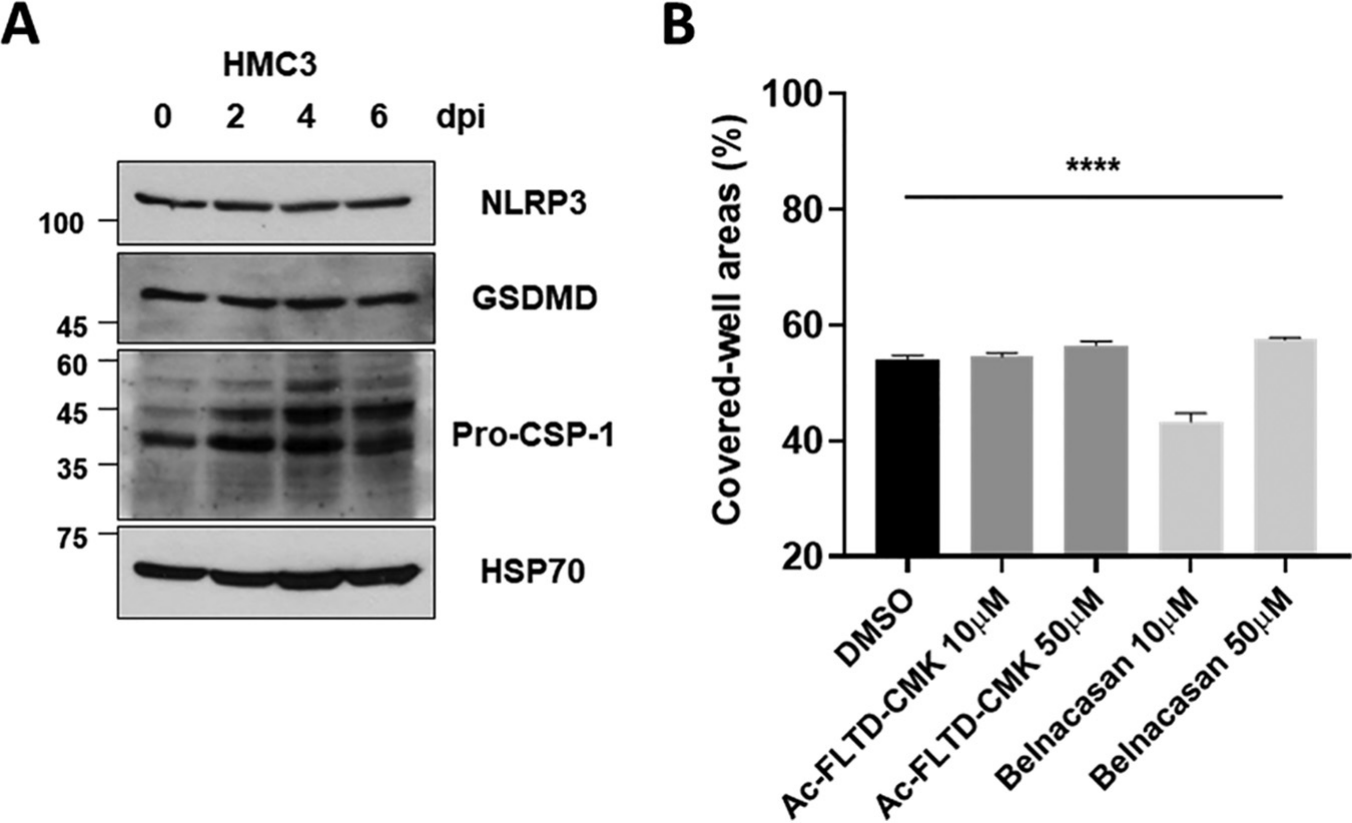
Pyroptosis might not be promoted by SARS-CoV-2 infection in HMC3. (A) After SARS-CoV-2 infection in HMC3, NLRP3, GSDMD, and caspase-1 proteins were analyzed by Western blotting. Hsp70 served as a loading control. (B) The SARS-CoV-2-infected HMC3 cells were treated with caspase-1 inhibitors (Ac-FLTD-CMK and Belnacasan) for 6 days at the indicated concentrations. The surviving cells were stained with crystal violet, and then the percent measurements of the stained cell covered areas were determined by the ImmunoSpot reader. Statistically significant differences between the groups were determined by one-way ANOVA; ****, *P* < 0.0001. Symbols represent means ± SEM.

Because RNA viruses, such as vesicular stomatitis virus (VSV), encephalomyocarditis virus (EMCV), and Zika virus (ZIKV), promote inflammatory cell death, such as pyroptosis, in most immune cells (42, 43), we assessed the possibility of pyroptosis induced by SARS-CoV-2 infection in HMC3. We observed no changes in the protein expression levels of the NLR family pyrin domain containing 3 (NLRP3), or the cleavage of Gasdermin D (GSDMD) and caspase-1, which suggests that pyroptosis might not have been promoted in SARS-CoV-2-infected HMC3 (Fig. 6A). The treatments of Ac-FLTD-CMK (caspase-1 inhibitor) and Belnacasan (VX-765, caspase-1 inhibitor) as described above also did not recover the cell viability (Fig. 6B). Taken together, these results suggest that SARS-CoV-2 induces cell death in HMC3 through both intrinsic and extrinsic death receptor-mediated apoptosis pathways.

### SARS-CoV-2 can infect microglia of K18-hACE2 mice leading to microgliosis and cell death.

**FIG 7:**
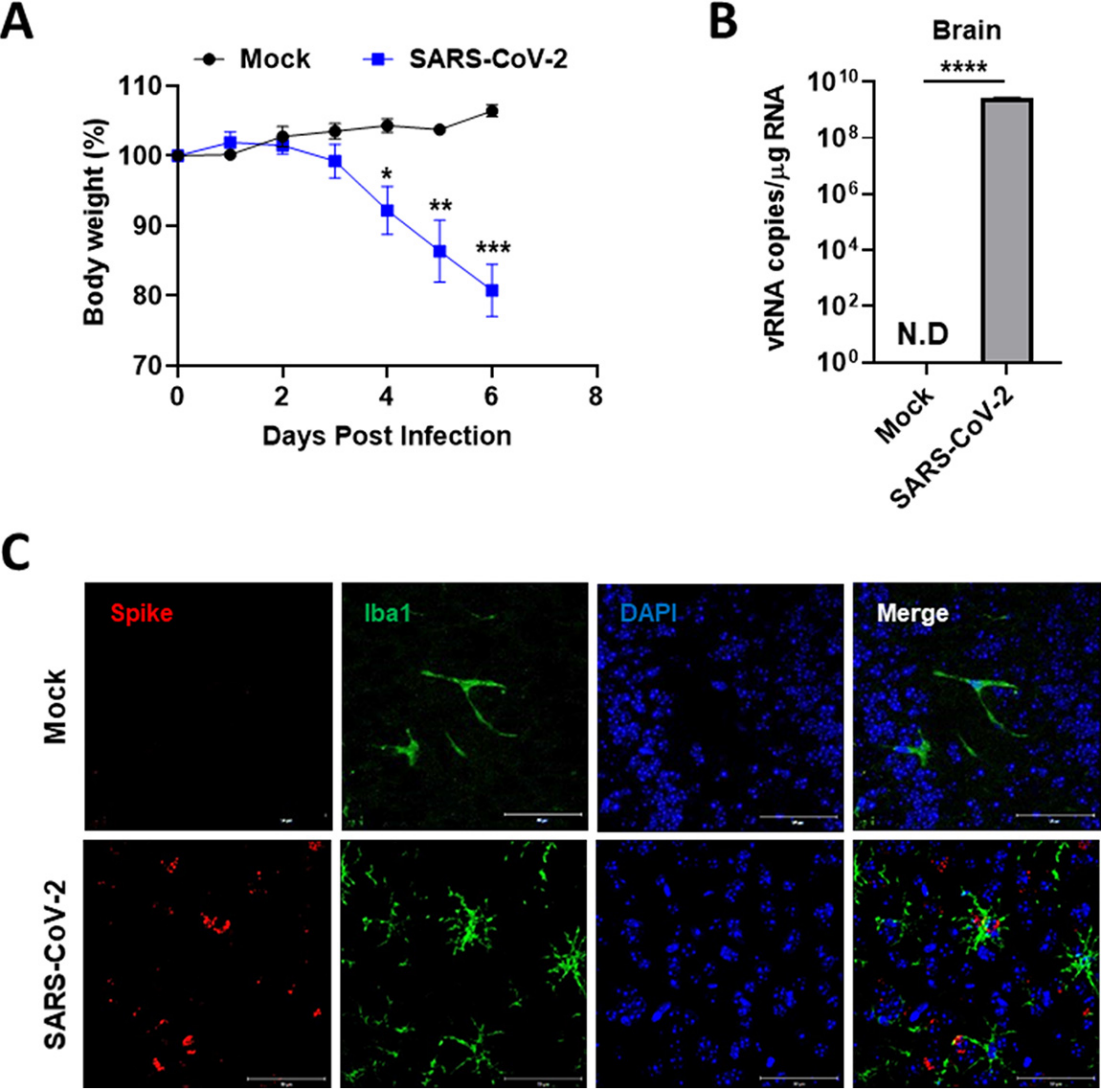
Microglia of K18-hACE2 mice were infected by intranasally administered SARS-CoV-2. (A) The SARS-CoV-2 inoculum (50 μL, 100 MLD_50_) was intranasally administered to a susceptible mouse model (K18-hACE2, *n* = 4). Their body weight was measured every day (Mock: black; Infected: blue). (B) At 6 d postinfection (dpi), the brain homogenates of the mock or infected mice were used to detect the viral RNA by quantitative real-time PCR (RT-qPCR), and the graph indicates viral RNA copies per microgram of total RNA. (C) The colocalization of SARS-Cov-2 spike protein and microglial Iba1 at 6 dpi in the infected mice by immunofluorescence staining. Scale bars = 50 μm. Statistically significant differences between the groups were determined by multiple Student's *t* test (A) and Student's *t* test (B); *, *P* < 0.05; **, *P* < 0.01; ***, *P* < 0.001; ****, *P* < 0.0001. Symbols represent mean ± SEM.

To substantiate this viral infection and microglial activation *in vivo* model, we used transgenic mice expressing the human ACE2, driven by the cytokeratin-18 gene promoter (K18-hACE2 mice), which were established for a model of SARS-CoV-2 infection and viral spread through the olfactory pathway (44–46). We inoculated 8-week-old heterozygous male K18-hACE2 mice via the intranasal route with 2 × 10^4^ plaque-forming units (PFU) of SARS-CoV-2. At 6 dpi infected mice showed a marked weight loss that was approximately 20% of their body weight (Fig. 7A). The viral RNA was detected in the brains of SARS-CoV-2 infected mice (Fig. 7B). Notably, the colocalization of SARS-CoV-2 spike protein (S) and Iba1, the most used marker of microglia, was detected mostly at 6 dpi in the brains (Fig. 7C). Another viral protein, NP, was also colocalized with Iba1 in the brains, confirming the previous findings (Fig. S6). From these results, we suggest SARS-CoV-2 infection of microglia in an *in vivo* model.

**FIG 8:**
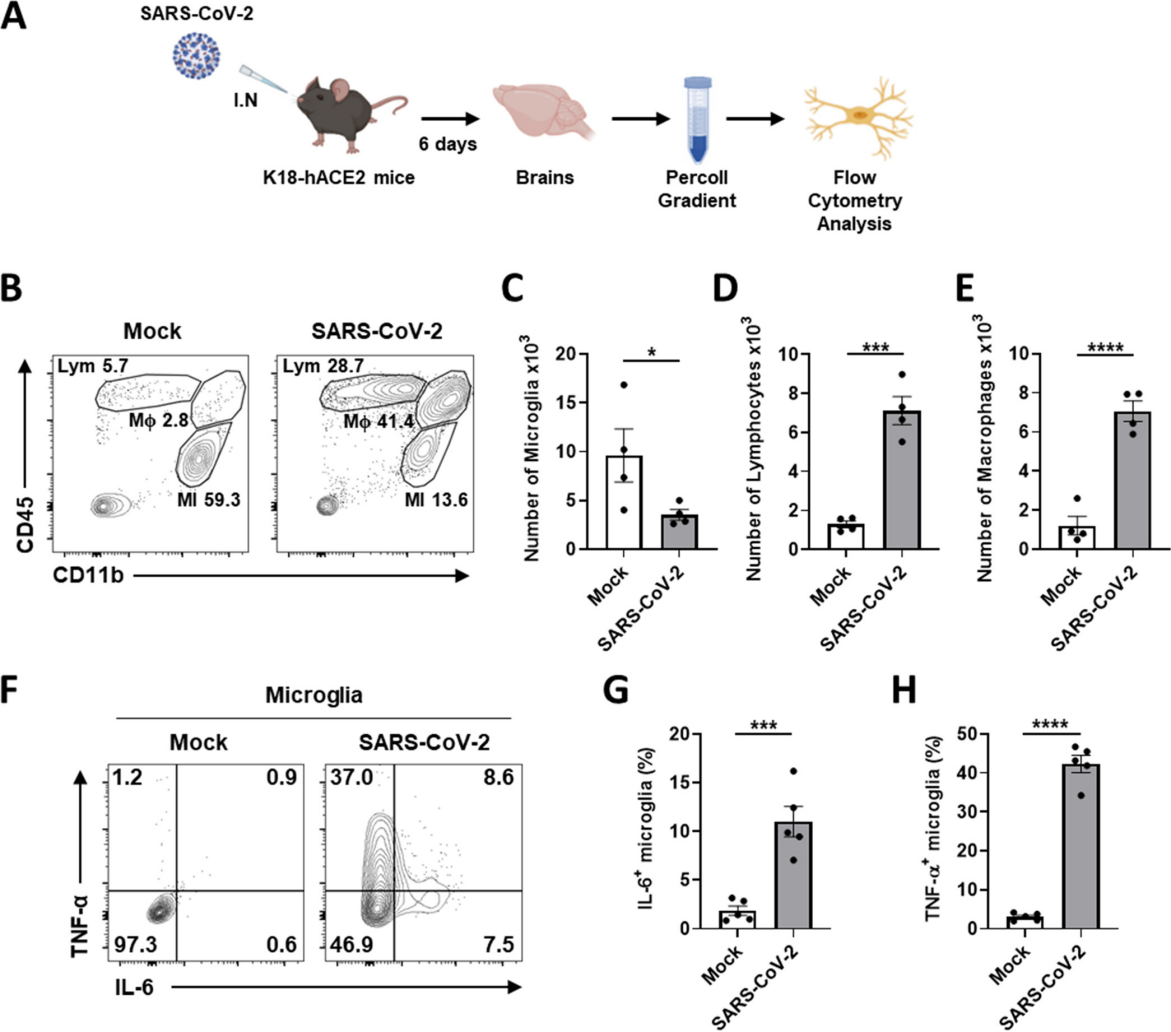
Microglial proinflammatory activation and depopulation by SARS-CoV-2 infection in K18-hACE2 mice. (A) Schematic of the experiment for B to H, created with BioRender.com. After 6 days, brains of mock or SARS-CoV-2-infected mice were extracted and used for Percoll gradient centrifugation to isolate mononuclear cells containing microglia for the flow cytometry analysis. The cellular surface of isolated mononuclear cells was stained with CD11b and CD45 antibodies. (B) Representative flow plot gated on leukocytes shows gating for microglia (MI, CD11b^+^, CD45^low^), macrophages (Mϕ, CD11b^+^, CD45^high^), and lymphocytes (Lym, CD11b^−^, CD45^high^). (C to E) Bar graphs show the number of microglia (C), lymphocytes (D), and macrophages (E) isolated per brain at 6 dpi. (F) Representative flow plot gated on microglia shows activated microglia with highly expressed IL-6 and TNF-α to separate activated from ramified microglia. (G and H) Bar graphs indicate the percentage of activated microglia, highly expressing IL-6 (G) and TNF-α (H). Statistically significant differences between the groups were determined using Student's *t* test; *, *P* < 0.05; ***, *P* < 0.001; ****, *P* < 0.0001. Symbols represent the mean ± standard error of the mean (SEM).

To demonstrate that SARS-CoV-2 infection of microglia induces proinflammatory activation following cell death in these mice, their brain homogenates were used to isolate leukocytes containing microglia by 30% and 70% Percoll discontinuous gradient centrifugation, followed by flow cytometry analysis with cell surface markers of microglia, CD11b and CD45, as illustrated in Fig. 8A The isolate consisted of three separated populations, including that of lymphocytes (CD11b^−^, CD45^high^), macrophages (CD11b^+^, CD45^high^), and microglia (CD11b^+^, CD45^low^) (47). While microglia account for most CNS leukocytes of uninfected mice, SARS-CoV-2 infection altered their population (Fig. 8B). Microglia were significantly depopulated by SARS-CoV-2 infection (Fig. 8C). On the other hand, the numbers of infiltrating lymphocytes and macrophages dramatically increased about seven times (Fig. 8D and E). From the microglia (CD11b^+^, CD45^low^) subgating, we made a plot that shows TNF-α and IL-6 (Fig. 8F). Most microglia were activated with the high expression of TNF-α and IL-6, compared to the resting microglia of uninfected mice (Fig. 8G and H). These data indicate that SARS-CoV-2 infection of microglia induces neuroinflammatory processes, such as microgliosis, immune cell infiltration, and cell death *in vivo*.

## DISCUSSION

Although COVID-19 is primarily known as a respiratory disease, symptoms related to changes in the peripheral nervous system have recently been reported in patients (15). There is evidence of viral invasion into the brain through the olfactory or vagal nerve and/or the oral and ophthalmic routes, with transsynaptic neuronal spread to other brain regions in COVID-19 patients’ autopsies and a SARS-CoV-2-infected mouse model (25, 29, 48). Microglia, expressing SARS-CoV-2 entry factors, including ACE2 and TMPRSS2 (30), can be infected by the invading SARS-CoV-2 and, subsequently, activated (49). Recently, emerging data have shown the presence of NP expression of Iba1-positive cells in the brains of SARS-CoV-2-infected hamsters and even COVID-19 patients with microgliosis (32, 50, 51). Activation of microglia could render individuals more at risk of developing neurological and psychiatric complications, even after complete clinical recovery from the infection (52). In addition, dysfunctional or aberrant microglial activity could severely impair cognitive functions, including judgment, decision making, learning, and memory (53). Hence, proinflammatory activation by SARS-CoV-2 infection of microglia might have critical outcomes on the short-, moderate-, or long-term neurological and psychiatric consequences of SARS-CoV-2 infection.

In this study, we found that SARS-CoV-2 directly infects a human microglial cell line. Regarding the RNA-seq analysis, it was observed that the viral infection-induced ER stress and immune responses in the early phase and apoptosis in the late phase. The microglia infected with SARS-CoV-2 were activated and polarized toward the M1 phenotype, the mediator of proinflammatory responses. In addition, SARS-CoV-2 infection triggered apoptosis as one of the CPEs in human microglia through both the intrinsic and extrinsic pathways, further provoking cell death. Indeed, SARS-CoV-2 has been reported to induce both intrinsic and extrinsic apoptosis in lung tissues and cell lines (54). More recently, SARS-CoV-2-encoded membrane glycoprotein (M) and NP have been demonstrated to trigger apoptosis (55), suggesting a mechanism of apoptosis in SARS-CoV-2-infected microglia. Moreover, we observed SARS-CoV-2 infection of microglia in K18-hACE2 mice, followed by an increase in the number of activated microglia and immune cell accumulation in the brain, and a decrease in the total number of microglia.

Given the colocalization of viral proteins and Iba1 in brains of patients with COVID-19 by postmortem studies (32, 51), we herein suggest a possibility that SARS-CoV-2 directly infects human microglia, inducing a CPE, which is cell death. In addition, human microglia could be one model to study viral pathogenesis. Microglia are important not only for the innate but also for the adaptive immune responses to pathogen infection of the brain (56). Depletion of microglia can lead to ineffective T cell responses by reduction of the total number and percentage of CD4^+^ and regulatory T cells. Consequently, the depletion of microglia causes increased viral replication and increased neurological manifestations in the brain (57, 58). Therefore, microglial cell death by SARS-CoV-2 infection might be linked to the lack of immune response and consequent increased viral replication, leading to increased neurological manifestations.

The activation of microglia and complement-mediated pathways, which leads to the synthesis of inflammation mediators, is the key element of main inflammatory neurological diseases (59). Many viruses, including HIV-1, herpes simplex virus (HSV), and ZIKV, infect microglia and cause neuroinflammation via microglial activation. C3 and its cleavage products attract microglia to gather around the neurons to exert phagocytotic activity and clear the presynaptic ends (60). The increase in the number of activated microglia via viral infection can have detrimental effects, indirectly by activating astrocytes (61) and T lymphocytes (62, 63), and directly by inducing neuronal damage and death, further contributing to neuronal degeneration (56, 64). In addition, the cytokine storm produced by microglia can lead to increased blood-brain barrier (BBB) breakdown and might be responsible for several neurological symptoms of COVID-19 (49). Thus, microglia could be a potential target for SARS-CoV-2 and could help the spread of the virus in the CNS. In future studies, to gain more insights into the SARS-COV-2 neuropathology in patients with COVID-19, it will be important to consider the relationship between SARS-CoV-2 and microglia. Taken together, our findings indicate that microglia are potential mediators of neurological diseases by SARS-CoV-2 and, consequently, can be targets of therapeutic strategies against neurological disorders in patients with COVID-19.

Our study has some limitations. Viral RNA detection and proinflammatory responses and CPE in iPSC-Microglia should have been evaluated to further evaluate susceptibility to SARS-CoV-2 infection of microglia; however, the long incubation time was a limitation in this investigation because iPSC-Microglia undergo the cell death after prolonged incubation post the microglial maturation. Another limitation is that we used K18-hACE2 mice to assess microglial susceptibility to SARS-CoV-2, rather than the more naturally sensitive to viral infection model, such as wild-type Syrian hamsters. While the expression and distribution of hACE2 are under the cytokeratin-18 gene promoter in K18-hACE2 mice, these mice have been widely used and have the advantage of investigating extrapulmonary replication of SARS-CoV-2.

## MATERIALS AND METHODS

### Cells and viruses.

Human microglial clone 3 (HMC3) (CRL-3304), Caco-2 (HTB-37), and Vero E6 (CRL-1586) cell lines were purchased from ATCC (Manassas, VA, USA). These cells were maintained in Eagle’s Minimum Essential Medium (EMEM; Welgene, Gyeongsangbuk-do, South Korea) containing 10% fetal bovine serum (FBS) (Gibco, Waltham, MA, USA) and 1% Pen/Strep (Gibco). iPSC-Microglia were derived from human induced pluripotent stem cells using a published protocol (65) with modifications. Primary human brain microglial cells (PHM) were purchased from Alphabioregen (PHM001, Boston, MA, USA). The SARS-CoV-2 Korean strain (GISAID accession no. MW466791), isolated from a patient in South Korea, was obtained from Korea Centers for Disease Control and Prevention (KCDC) and propagated in Vero cells (CCL-81, ATCC).

Cells (1 × 10^5^ cells per well) were plated into six-well plates and inoculated with one multiplicity of infection (MOI) SARS-CoV-2 in EMEM containing 2% FBS on the next day. After incubation for 1 h, the inoculum was removed, and the cells were washed two times. The medium was changed to EMEM containing 10% FBS. At 2, 4, or 6 dpi, the total cellular RNA was extracted using the RNeasy Minikit (Qiagen, Hilden, Germany). For iPSC-Microglia and PHM cells, 2 × 10^4^ cells per well were plated into 24-well plates and infected with one MOI SARS-CoV-2 as described before, followed by detection of viral RNA after 24 h of incubation.

For the virus neutralization, the inoculum was further incubated with a CR3022 neutralizing antibody (ab273073, Abcam, Cambridge, UK) for 1 h. For crystal violet staining, cells were stained with 0.5% crystal violet staining solution in 25% methanol for 30 min. The plates were then washed three times with water and dried. The cell-covered areas were measured by the ImmunoSpot reader (CTL, Shaker Heights, OH, USA). The caspase inhibitors used in this study, including Z-DEVD-FMK (S7312), Z-VAD-FMK (S7023), Z-IETD-FMK (S7314), and Belnacasan (VX-765, S2228), were purchased from Selleckchem (Houston, TX, USA).

### Biosafety.

All procedures were performed in a biosafety level 3 (BSL3) or animal BSL3 facility for SARS-CoV-2-related experiments, with approval from the Korea Research Institute of Chemical Technology (KRICT) and by personnel equipped with powered air-purifying respirators.

### Mice.

Eight-week-old male B6.Cg-Tg (K18-hACE2) 2Prlmn/J mice were purchased from the Jackson Laboratory and maintained in a biosafety level 2 (BSL2) animal facility in the Korea Research Institute of Chemical Technology (KRICT). All protocols were approved by the Institutional Animal Care and Use Committee (IACUC; protocol no. 8A-M6, 2021-8A-02-01, and 2021-8A-03-03).

Virus inoculations (SARS-CoV-2; 2 × 10^4^ PFU) were performed by the intranasal route (I.N.) under anesthesia using isoflurane in a BSL3 animal facility, and all efforts were made to minimize animal suffering. Body weights were measured every day postinfection.

### Microglia isolation.

Isolation of microglia in mice was performed following the protocols previously described (66, 67), with minimal modifications. Mock or SARS-CoV-2 infected mice were anesthetized by isoflurane, followed by perfusion with 10 or 20 mL of cold 1× DPBS (Gibco) into the left ventricle to remove blood from the tissues. Brains were transferred to a six-well plate containing cold Hanks’ Balanced Salt Solution (HBSS) (Gibco) and the plates were kept on ice. The generation of brain cell suspension by a 70-μm pore sized-cell strainer (SPL, Gyeonggi-do, South Korea) was made in 10 mL per brain of digestion cocktail containing 0.5 mg/mL DNase I (Roche, Basel, Switzerland) and 1 mg/mL Collagenase A (Roche) in HBSS. The suspension was incubated at 24°C for 30 min, followed by centrifugation for 7 min at 300 × *g*, 18°C. The cell pellet was resuspended with 30% Percoll (Sigma-Aldrich, St. Louis, MO, USA) in HBSS, and then was slowly layered over 70% Percoll in HBSS in a 15 mL-conical tube. About 2 mL of interphase volume was collected to a new tube after gradient centrifugation for 40 min at 200 × *g*, 18°C. Isolated mononuclear cells were washed three times in a volume of 500 μL of HBSS containing 0.01 M HEPES (Gibco), using a microcentrifuge for 7 min at 600 × *g*, 4°C.

### Flow cytometry analysis.

Isolated brain mononuclear cells from the mock or SARS-CoV-2-infected mice in cell staining buffer (phosphate-buffered saline [PBS] with 1% FBS and 0.09% NaN_3_) were stained for 30 min with fluorescence-conjugated antibodies, namely, brilliant violet 421 anti-mouse/human CD11b antibody (101236, BioLegend, San Diego, CA, USA), PE/Cyanine7 anti-mouse CD45 antibody (103114, BioLegend), APC anti-mouse TNF-α antibody (506307, BioLegend), FITC anti-mouse IL-6 monoclonal antibody (MP5-20F3) (11-7061-82, eBioscience, San Diego, CA, USA), FITC anti-human ACE2 antibody (NBP2-7211F, Novus Biologicals, Centennial, CO, USA), and Alexa 647 anti-Iba1 antibody (78060S, Cell Signaling Technology, Danvers, MA, USA). Cells were then analyzed by FACSAria III sorter (BD Biosciences, San Jose, CA, USA), and data were analyzed by FlowJo software (BD Biosciences). All fluorochromes were compensated. The total leukocyte population was gated for microglia (CD11b^+^, CD45^low^). For annexin V staining, mock or SARS-CoV-2-infected HMC3 were stained with FITC-recombinant human annexin V, following the protocols of Annexin V-FITC Apoptosis Detection kit (BMS500FI-20, eBioscience).

### RT-qPCR.

Quantitative RT-PCR (QuantStudio 3, Applied Biosystems, Foster City, CA, USA) was performed with a one-step Prime script III RT-qPCR mix (RR600A, TaKaRa, Kyoto, Japan). The viral RNA of NP was detected by the 2019-nCoV-N1 probe (catalog number 10006770, Integrated DNA Technologies, Coralville, IA, USA). The IL-1β, IL-6, IL-12, TNF-α, IFN-β, IFN-λ1, NOS2, and Arginase-1 genes were detected by individually customized probes (Integrated DNA Technologies).

For RT-PCR analysis of GPR34, MERTK, and P2RY12, reverse transcription was carried out using GoScript Reverse Transcription System (Promega) according to the manufacturer’s instructions. The PCR amplifications were performed in SYBR green PCR master mix and CXR (Applied Biosystems, Warrington, U.K.) including primers using an ABI 7500 (Applied Biosystems). Average threshold cycle (Ct) values of GPR34, MERTK, and P2RY12 from triplicate PCRs were normalized from average Ct values of GAPDH.

### Focus forming assay.

The cell culture medium was serially diluted in EMEM containing 2% FBS and was added to 4 × 10^4^ Vero E6 cells plated on 96-well plates. After incubation for 8 h at 37°C, cells were washed and fixed with a 4% formaldehyde solution. Cells were stained with the anti-SARS-CoV-2 NP antibody (40143-R001, Sino Biological, Beijing, China) and a secondary horseradish peroxidase-conjugated goat anti-rabbit IgG (Bio-Rad, Hercules, CA). The signal was developed using an insoluble tetramethylbenzidine (TMB) substrate (Promega, Madison, WI, USA), and the number of infected cells was counted using an ImmunoSpot reader (CTL, Shaker Heights, OH).

### RNA-seq and analysis.

A sequencing library was prepared with TruSeq Stranded mRNA Sample Prep kit and sequenced on NovaSeq 6000 (Illumina, San Diego, CA, USA), yielding more than 6G bases of sequences for each sample. From the sequenced reads, adaptor sequences were removed using Cutadapt (version 3.1) (68) and aligned to the hybrid reference genomes of humans (GRCh38.p13_ENS100) and SARS-CoV-2 (ASM985889_v3) with STAR aligner (version 2.7.6a) (69). Aligned reads were quantified at the gene level by HTSeq (version 0.13.5) (70) with “intersection-nonempty” mode. Genes with lower than five counts for the total count per gene were removed for further analyses. DEG analysis was processed with DESeq2 (version 1.30.1) (71) using abs (log_2_ change) > 1 and adjusted *P* value (Benjamini-Hochberg) < 0.01 as the cutoff. Multidimensional scaling analysis was performed with the clustermap function in Python seaborn package (version 0.11.1) using genes with mean FPKM > 1 among the samples and transformed to log_2_(FPKM + 1). Overrepresentation analysis of the DEGs enriched to GO Biological Process 2018 with EnrichR (72) with adjusted *P* value (Benjamini-Hochberg) <0.05 as the cutoff. Network analysis was presented by Metascape (73) using the GO Biological Process gene sets.

### Immunofluorescence assay.

After transcardial perfusion with cold 4% paraformaldehyde in PBS, brain tissues of SARS-CoV-2 infected K18-hACE2 mice were dissected and fixed by immersion in 4% paraformaldehyde in PBS overnight at 4°C. The brain sections (30 μm thickness) were permeabilized with 0.2% Triton X-100 in 1% BSA/PBS for 30 min, washed in PBS, and blocked with 0.5% BSA in PBS for 15 min, followed by incubation overnight at 4°C with primary antibodies, namely, anti-SARS-CoV-2 S (40150-T62-COV2, Sino Biological), and anti-Iba1/AIF1 (MABN92, Merck Millipore, Burlington, MA, USA). After washing twice, further incubation was carried out with Alexa Fluor 488-conjugated anti-rabbit antibody (A32731, Thermo Fisher Scientific, Waltham, MA, USA) and Alexa Fluor 594-conjugated anti-mouse antibody (A32744, Thermo Fisher Scientific). Immunofluorescence was observed by confocal microscopy (LSM700, Carl Zeiss, Oberkochen, Germany).

For the cell lines, the SARS-CoV-2-infected cells were fixed with 4% paraformaldehyde in PBS overnight at 4°C and then permeabilized with 0.5% Triton X-100 in PBS for 10 min, followed by washing thrice with PBS. Blocking buffer (0.1% Tween 20, 1% bovine serum albumin [BSA] in PBS) was added to remove the nonspecific binding. Cells were immunostained overnight at room temperature with primary antibodies, namely, anti-dsRNA J2 (MABE1134, Sigma-Aldrich), anti-SARS-CoV-2 NP (40143-R019, Sino Biological), anti-SARS-CoV-2 S (40150-T62-COV2, Sino Biological), and anti-CD68 (sc-17832, Santa Cruz Biotechnology, Dallas, TX, USA). After washing thrice, further incubation was carried out with Alexa Fluor 488-conjugated anti-rabbit antibody (A32731, Thermo Fisher Scientific) and Alexa Fluor 594-conjugated anti-mouse antibody (A32744, Thermo Fisher Scientific). Immunofluorescence was observed by confocal microscopy.

### ELISA.

The culture supernatants were collected from infected cells and used for the detection of IL-1β, IL-6, IL-10, and TNF-α. Each cytokine was determined by the corresponding ELISA kit (IL-1β, K0331800; IL-6, K0331194; IL-10, K0331123; TNF-α, K0331131; Komabiotech, Seoul, South Korea), following the manufacturer’s instructions.

### Western blotting.

Cells were lysed in radioimmunoprecipitation (RIPA) buffer (Thermo Fisher Scientific), and proteins in the lysate were separated in a denaturing polyacrylamide gel and transferred to a polyvinylidene fluoride (PVDF) membrane (Merck Millipore, Burlington, MA, USA). The membrane was incubated with 5% skim milk (BD Biosciences) in tris-buffered saline with 0.1% Tween 20 (TBST) buffer and the primary antibodies, namely, anti-SARS-CoV-2 NP (40143-R001, Sino biological), anti-CD68 (sc-17832, Santa Cruz Biotechnology) anti-GSDMDC1 (sc-81868, Santa Cruz Biotechnology), anti-Actin (sc-47778, Santa Cruz Biotechnology), anti-Hsp70 (sc-24, Santa Cruz Biotechnology), anti-CX3CL1 (ab25088, Abcam), anti-CX3CR1 (ab8021, Abcam), anti-CD16 (80006S, Cell Signaling Technology), anti-phospho-Stat1 (9167S, Cell Signaling Technology), anti-Stat1 (14994S, Cell Signaling Technology), anti-Fas (4233T, Cell Signaling Technology), anti-DR4 (42533T, Cell Signaling Technology), anti-DR5 (8074T, Cell Signaling Technology), anti-TNFR2 (3727T, Cell Signaling Technology), anti-Bcl-2 (4223T, Cell Signaling Technology), anti-Bim (2933T, Cell Signaling Technology), anti-Bid (2002T, Cell Signaling Technology), anti-Bax (5023T, Cell Signaling Technology), anti-caspase-9 (9502S, Cell Signaling Technology), anti-caspase-8 (9746S, Cell Signaling Technology), anti-caspase-3 (9665S, Cell Signaling Technology), anti-caspase-1 (3866S, Cell Signaling Technology), anti-NLRP3 (15101S, Cell Signaling Technology), and anti-PARP (9542S, Cell Signaling Technology). Horseradish peroxidase (HRP)-conjugated secondary antibodies from Bio-Rad and ECL reagents (Thermo Fisher Scientific) were used for protein detection.

### Statistical analysis.

All experiments were performed at least three times. All data were analyzed using GraphPad Prism 8.0 software (GraphPad Software, San Diego, CA, USA). *P* < 0.05 was considered statistically significant. Specific analysis methods are described in the figure legends.
