# Supplementary material for: SARS-CoV-2 Infection of Microglia Elicits Proinflammatory Activation and Apoptotic Cell Death
Source: Microbiol Spectr. 2022 May 5;10(3):e01091-22. doi: 10.1128/spectrum.01091-22 (PMC9241873; doi:10.1128/spectrum.01091-22)
Supplement: SUPPLEMENTAL FILE 1 — Supplemental material. Download spectrum.01091-22-s001.pdf, PDF file, 1.9 MB [file spectrum.01091-22-s001.pdf]

**Supplementary Information**

# SARS-CoV-2 Infection of Microglia Elicits Pro-inflammatory Activation and Apoptotic Cell Death

Gi Uk Jeong[a], Jaemyun Lyu[b], Kyun-Do Kim[a], Young Cheul Chung[c], Gun Young Yoon[a], Sumin Lee[a], Insu Hwang[a], Won-Ho Shin[c], Junsu Ko[b], June-Yong Lee[d], and Young-Chan Kwon[a]#

[a]Center for Convergent Research for Emerging Virus Infection, Korea Research Institute of Chemical Technology, Daejeon 34114, Republic of Korea

[b]Arontier Co., Ltd. Seoul 06735, Republic of Korea

[c]Department of Predictive Toxicology, Korea Institute of Toxicology, Daejeon 34114, Republic of Korea

[d]Department of Microbiology and Immunology, College of Medicine, Yonsei University, Seoul 03722, Republic of Korea

#Correspondence: yckwon@krict.re.kr; Tel: +82-42-860-7028

## Supplementary figures, legends, and table

## Supplementary Fig. 1

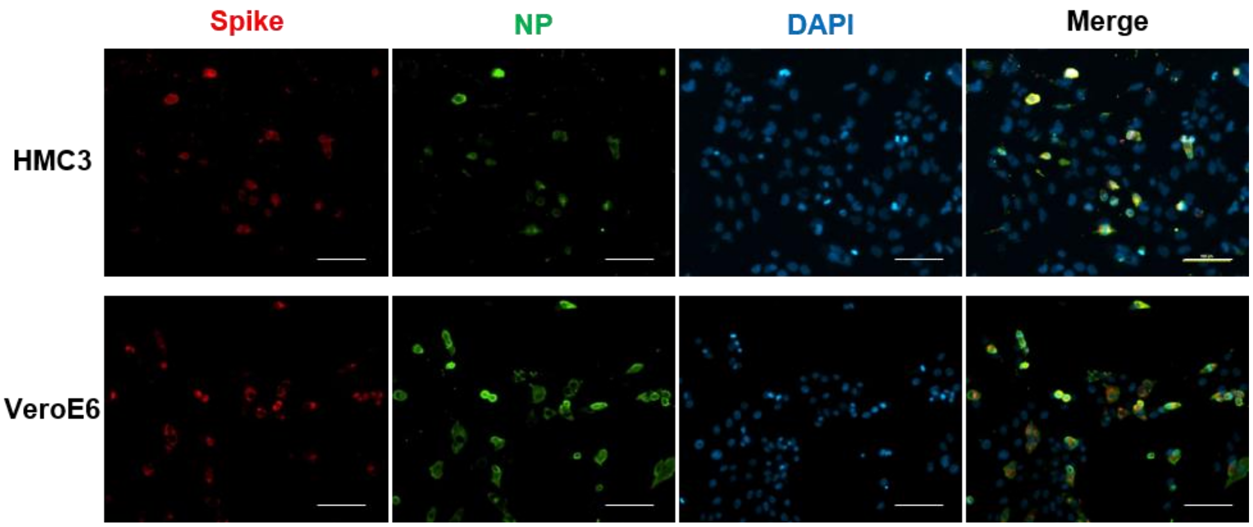


**Supplementary Fig. 1**. **Fluorescence microscope images of the viral protein expression (S and NP) in SARS-CoV-2 infected HMC3.** Representative images of infected HMC3 by confocal microscopy. Scale bars = 100 µm.

# Supplementary Fig. 2

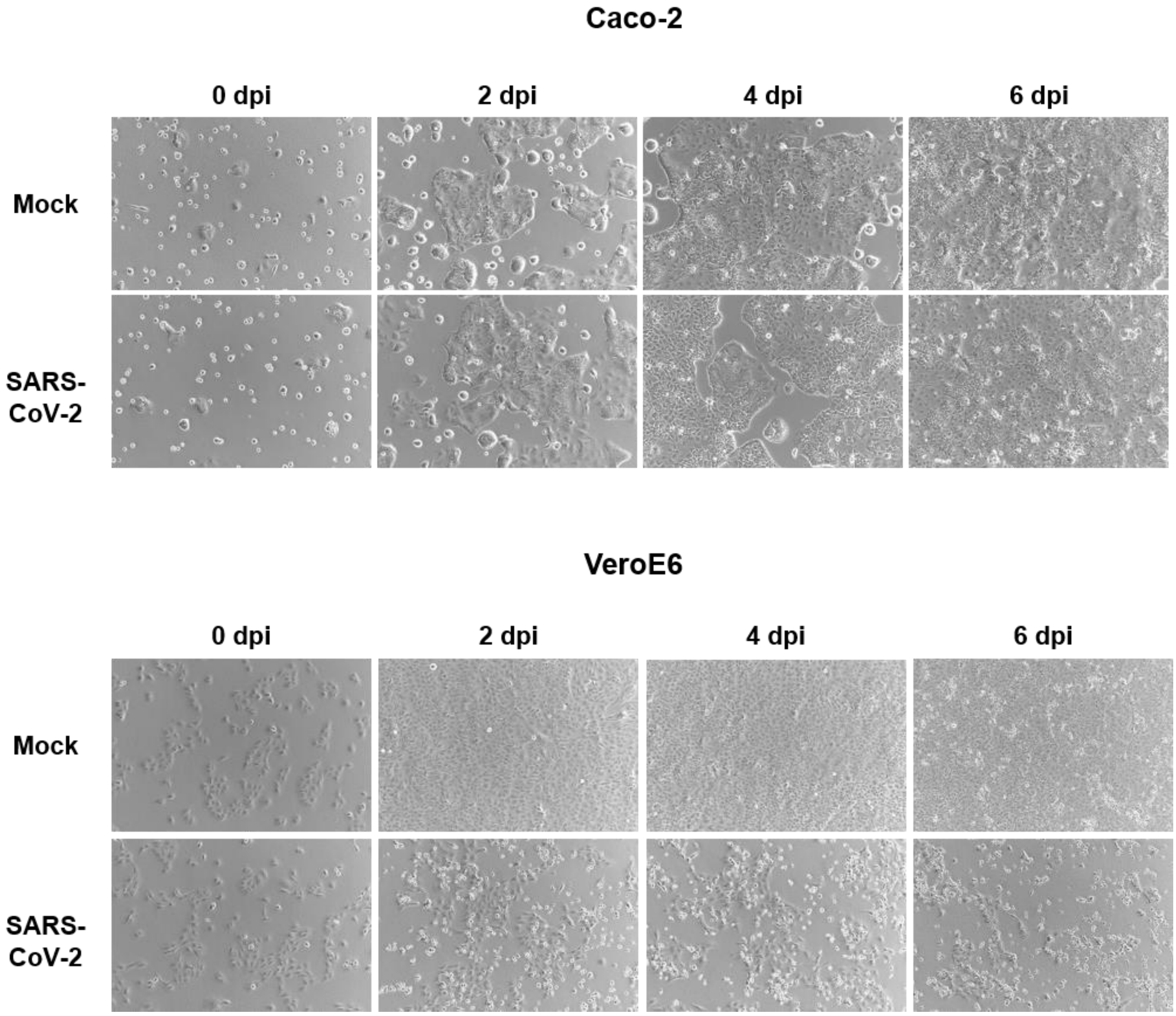


**Supplementary Fig. 2. Time-course cell images of Caco-2 and Vero E6 after SARS-CoV-2 infection.** Phase-contrast images of infected Caco-2 and Vero E6 by microscopy.

Supplementary Fig. 3

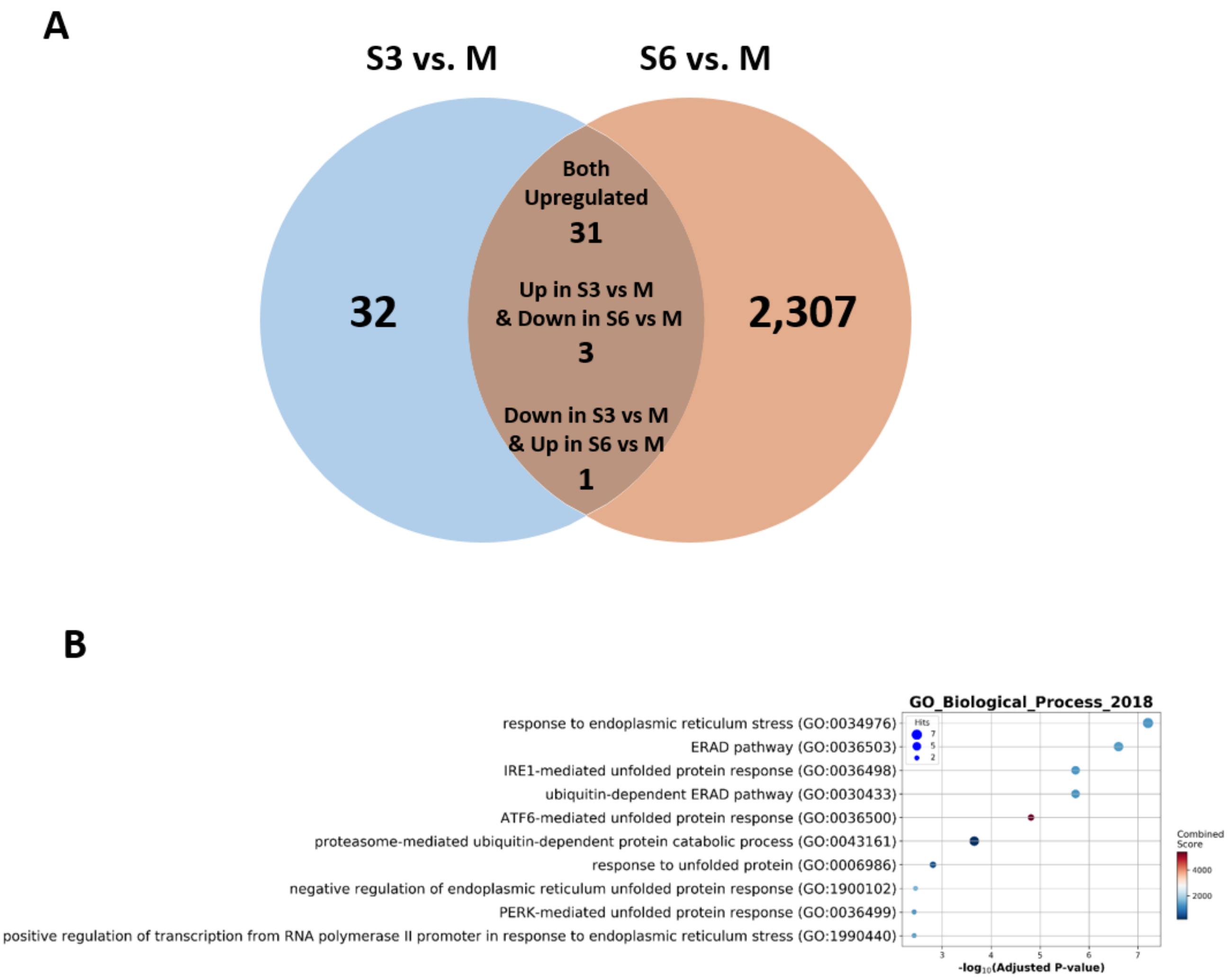


**Supplementary Fig. 3**. **Gene count combinations in overlapping differentially expressed genes (DEGs) and over-representation analysis (ORA) of upregulated DEGs at both 3 dpi (S3) and 6 dpi (S6) compared to mock (M).** (A) Overlapping DEGs displayed in this Venn diagram were classified by up- or downregulated genes at S3 and S6. (B) ORA of gene ontology biological process terms using upregulated DEGs at both S3 and S6.

Supplementary Fig. 4

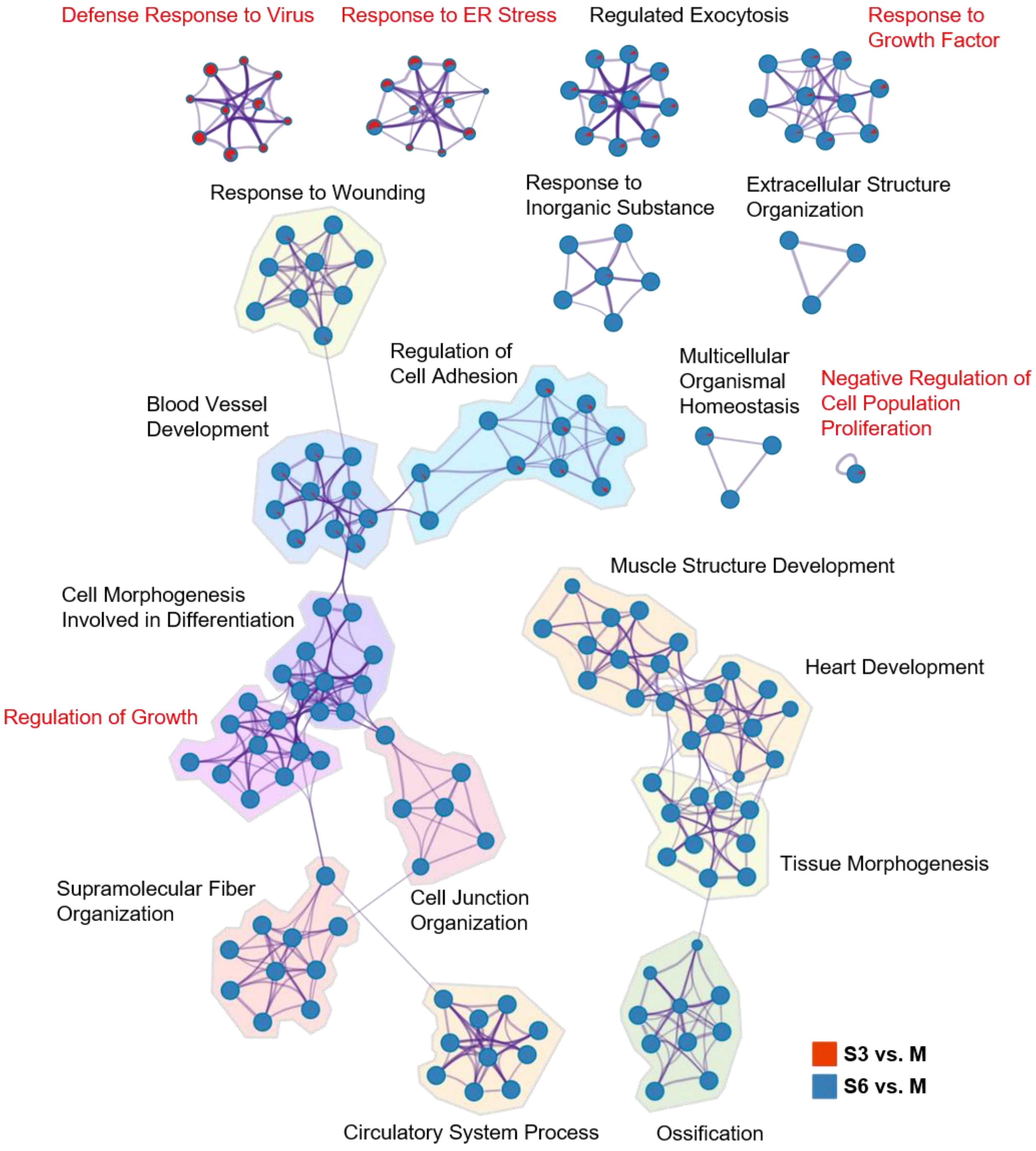


**Supplementary Fig. 4. A network of the significant changes in the gene ontology (GO) terms of SARS-CoV-2-infected HMC3 at 3 dpi (S3) and 6 dpi (S6) compared to that of mock (M).**

# Supplementary Fig. 5

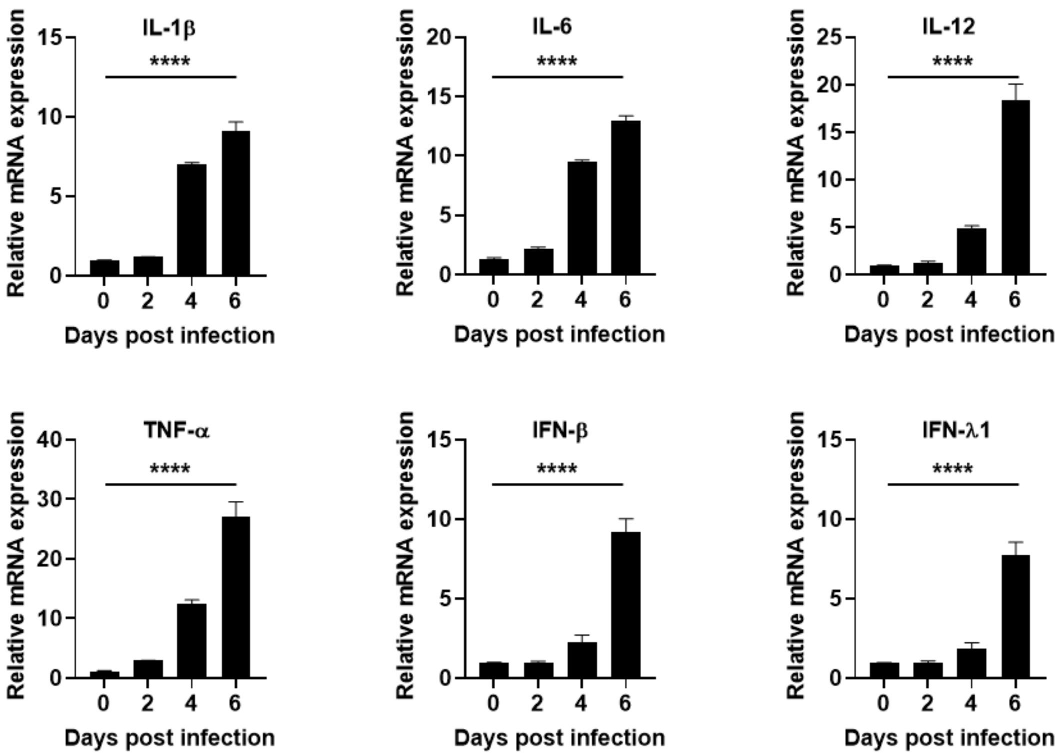


**Supplementary Fig. 5. Time-course expression pattern of interferons and pro-inflammatory cytokines during SARS-CoV-2 infection in HMC3.** RNA expression levels of interferons, IFN-β, and IFN-λ1, and pro-inflammatory cytokines, including IL-1β, IL-6, and IL-12, were analyzed by RT-qPCR after SARS-CoV-2 infection. Symbols in the bar graphs represent means ± SEM. Statistically significant differences between the groups were determined by one-way analysis of variance (ANOVA); ****, $P < 0.0001$.

Supplementary Fig. 6

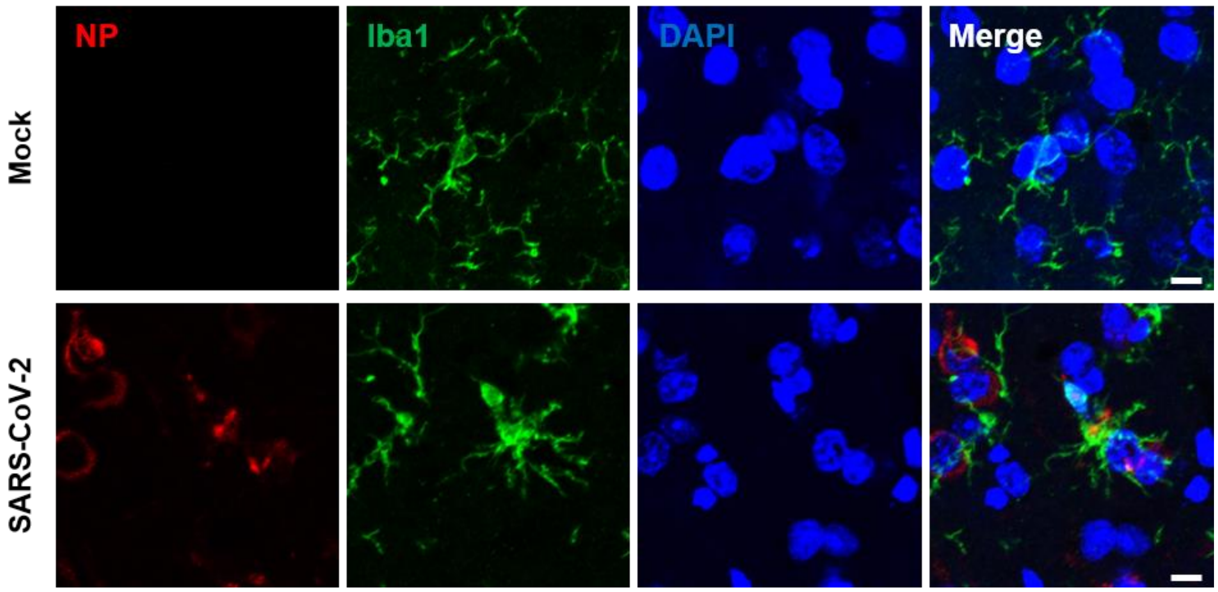


**Supplementary Fig. 6. Fluorescence microscope images of viral NP and microglial Iba1 in the brains of SARS-CoV-2-infected K18-hACE2 mice by confocal microscopy.** Scale bars = 10 μm.

**Table S1. The qPCR probes and primers sequences used in this study.**

| Gene | Forward (5'-3') | Reverse (5'-3') | Probe (5'-3') |
|---|---|---|---|
| NOS2 | CACCATCCTCTTTGCGACA | GCAGCTCAGCCTGTACT | 56-FAM/TATTCAGCT/ZEN/GTGCCTTCAACCCCA/3IABkFQ |
| ARG1 | ACTCCACTGACAACCACAAG | TGGCAGATATACAGGGAGTCA | 56-FAM/CCTTCAGGA/ZEN/GGAAAGATACAGGTTGTCCA/3IABkFQ |
| IL-1β | CAGCCAATCTTCATTGCTCAAG | GAACAAGTCATCCTCATTGCC | 56-FAM/AGAAGTACC/ZEN/TGAGCTCGCCAGTGA/3IABkFQ |
| IL-6 | GCAGATGAGTACAAAAGTCCTGA | TTCTGTGCCTGCAGCTTC | 56-FAM/CAACCACAA/ZEN/ATGCCAGCCTGCT/3IABkFQ |
| IL-12 | CAGTTATTGATGAGCTGATGCAG | CATGAAGAAGTATGCAGAGCTTG | 56-FAM/TGCCACAAA/ZEN/AATCCTCCCTTGAAGAAC/3IABkFQ |
| TNF-α | TGCACTTTGGAGTGATCGG | TCAGCTTGAGGGTTTGCTAC | 56-FAM/AGATGATCT/ZEN/GACTGCCTGGGCC/3IABkFQ |
| IFN-β | GAAACTGAAGATCTCCTAGCCT | GCCATCAGTCACTTAAACAGC | 56-FAM/TGAAGCAAT/ZEN/TGTCCAGTCCCAGAGG/3IABkFQ |
| IFN-λ1 | GGTTCAAATCTCTGTCACCACA | GAAGACAGGAGAGCTGCAAC | 56-FAM/TCAAGAAGG/ZEN/CCAGGGACGCC/3IABkFQ |
| β-actin | ACAGAGCCTCGCCTTTG | CCTTGCACATGCCGGAG | 56-FAM/TCATCCATG/ZEN/GTGAGCTGGCGG/3IABkFQ |
